# Mechanisms and Clinical Applications of Genome Instability in Multiple Myeloma

**DOI:** 10.1155/2015/943096

**Published:** 2015-10-22

**Authors:** Antonia Cagnetta, Davide Lovera, Raffaella Grasso, Nicoletta Colombo, Letizia Canepa, Filippo Ballerini, Marino Calvio, Maurizio Miglino, Marco Gobbi, Roberto Lemoli, Michele Cea

**Affiliations:** Hematology Unit, Department of Internal Medicine (DiMI), University of Genoa, IRCCS AOU San Martino-IST, 1600 Genoa, Italy

## Abstract

Ongoing genomic instability represents a hallmark of multiple myeloma (MM) cells, which manifests largely as whole chromosome- or translocation-based aneuploidy. Importantly, although it supports tumorigenesis, progression and, response to treatment in MM patients, it remains one of the least understood components of malignant transformation in terms of molecular basis. Therefore these aspects make the comprehension of genomic instability a pioneering strategy for novel therapeutic and clinical speculations to use in the management of MM patients. Here we will review mechanisms mediating genomic instability in MM cells with an emphasis placed on pathogenic mutations affecting DNA recombination, replication and repair, telomere function and mitotic regulation of spindle attachment, centrosome function, and chromosomal segregation. We will discuss the mechanisms by which genetic aberrations give rise to multiple pathogenic events required for myelomagenesis and conclude with a discussion of the clinical applications of these findings in MM patients.

## 1. Introduction

Multiple myeloma (MM) is a clonal B-cell malignancy characterized by excessive bone marrow plasma cells in association with monoclonal protein [1, 2]. The therapeutics currently available improve patients' survival and quality of life, but resistance to therapy and disease progression remain unsolved issues [3, 4]. Therefore, the definition of novel targeted vulnerabilities in MM biology remains a major basic and clinical research goal. Recent studies have demonstrated that MM is characterized by a significant heterogeneity, which is mainly related to molecular characteristics of the tumor clone [5]. Such feature, occurring also at early stages, makes MM quite different from other hematologic diseases such as leukemia and lymphomas that harbor a restricted number of genetic changes. By contrast, a wide variety of chromosomal and genomic rearrangements are frequently observed in solid tumors. Thus, MM is considered in between these two genetic landscapes with a complex oncogenic network deregulation [6].

Genome instability, defined by higher rate of genomic changes acquisition per cell division compared to normal cells, represents a prominent feature of MM cells [7]. There are various forms of genetic instability such as chromosomal instability (CIN), microsatellite instability (MSI), and base-pair mutations. CIN refers to the high rate by which chromosome structure and number changes in MM cells compared with normal cells. Numerical chromosome abnormalities may be generated by centrosome amplification or alterations in the spindle assembly checkpoint [8]. In contrast, structural alterations, such as chromosomal deletions or translocations, might arise from alterations in the repairing of DNA double strand breaks (DSBs). The specific contribution of each event in MM tumorigenesis is not fully understood, but the most frequently observed changes include hyperdiploidy [9], loss of chromosome 13 [10, 11], and specific translocation like t(11;14) (q13;q32); t(4;14)(p16;q32); or t(14;16)(q23;q32) [12–15]. Such aneuploidy can be interpreted as a consequence of the general chaos that progressively envelops cancer cells as they advance toward highly malignant states, or it is an inherent element of tumorigenesis. Indeed, in absence of the increased mutability associated with aneuploidy, most clones of incipient tumor cells could never succeed in acquiring all genetic alterations needed to complete multistep tumorigenesis. Therefore, cancer cells by changing their genomes through chromosome instability create promising configurations that allow growth of neoplastic cells. Although CIN represents the most common form of genomic instability, others have also been described including microsatellite instability, characterized by the expansion or contraction of the number of oligonucleotide repeats present in microsatellite sequences, and the base-pair mutations which refer to increased frequencies of base-pair mutations in tumor cells [7]. Overall, the comprehensive karyotypic analysis provides insights into molecular mechanisms and clinical management of MM. Indeed, chromosomal aberrations allow identifying two broad subtypes of disease, one characterized by chromosomal gains (hyperdiploidy) and the other by structural changes (nonhyperdiploidy), leading to different results in terms of prognosis [9].

However, causes of genomic instability remain to date unclear thus failing identification of universal driver event in MM cells. An increased c-MYC expression, K-RAS mutations and fibroblast growth factor receptor-3 (FGFR3) overexpression seem to be the most frequently genetic aberration observed during disease progression [16]; nevertheless additional genetic abnormalities further contribute to increase genetic complexity of such a tumor. It follows that MM genome is extremely heterogeneous with marked changes affecting both prognostic stratification and therapeutic approaches. In addition to this inter-MM heterogeneity, deep genome sequencing studies proved existence of intraclonal diversity affecting MM patients individually with altered clones present at diagnosis and during disease evolution [17–19]. Accordingly, genetic instability by supporting mutations development hugely increases complexity of MM, by allowing survival advantage and progression.

Based on these findings, here we will review the significance of this heterogeneity in MM cells, by focusing on biological relevance of genomic instability, and examining how the currently available therapeutic strategies can exploit this feature.

## 2. Heterogeneity of MM

A hallmark of almost all human cancers is represented by aberrations in their genomic architecture, which refers to permanent or temporary changes [18]. Among these alterations, CIN (gain or loss of whole chromosomes as well as inversions, deletions, duplications, and translocations of large fragments of chromosomes) is frequently observed in numerous solid tumors. As such this abnormality results in large-scale changes of genes, which are involved in cellular processes critical for maintenance of genome integrity during disease progression [20]. Based on these findings the two categories identified,* hyperdiploid* and* nonhyperdiploid*, show different prognostic significance with the latter associated with poorer overall survival (Table 1). Specifically, trisomies of chromosomes 3, 5, 7, 11, 15, 19, and 21 define hyperdiploid karyotype (50–60% of MM patients); otherwise the nonhyperdiploid karyotype is frequently characterized by translocations affecting immunoglobulin heavy chain (IGV) locus at 14q32, including t(11;14) and t(4;14), which are the most clinically relevant. Indeed t(11;14) is observed in 20% of MM patients and confers favorable outcome [11, 21]. Differently, t(4;14) occurs in 15% of MM patients and is associated with very poor prognosis with its presence requiring specific therapeutic approaches such as proteasome inhibitors or immunomodulatory agents [5]. Molecularly t(4;14) results in simultaneous overexpression of two genes located on 4p: the multiple myeloma SET domain (MMSET), which is a homologous of histone methyltransferase, and the fibroblast growth factor receptor 3 (FGFR3), which is an oncogenic receptor tyrosine kinase. As both genes have potential oncogenic activity, their deregulation triggered by chromosomal aberration is associated with poor survival. In general t(4;14), t(14;16), chromosome 13 deletion, and loss of 17p13 are associated with poor prognosis in patients undergoing high-dose therapy, whereas hyperdiploidy and t(11;14) translocations are associated with better outcome. One less frequent (6-7% of MM patients) but clinically relevant translocation is t(14;16), which involves MAF genes and confers poor outcome [22]. del(17p) is carried by 8–10% of patients and represents the most important aberration for prognosis since its presence is associated with a remarkable short survival irrespective of treatment [23]. Finally, several reports have shown additional abnormalities such as amp(1q), del(1p), del(12p), del(16q), and del(6q) having prognostic relevance, with the latter associated with worse prognosis then del(17p) [24, 25].

A large number of chromosomal changes including MYC translocations, loss or deletion of chromosome 13, deletions and/or amplifications of chromosome 1, and deletion of chromosome 17, are observed also during MM progression [18]. Indeed, about 45% of MM patients with advanced disease carry translocations and/or amplifications of the oncogene MYC that is associated with more aggressive disease. Also deletion of 17p13 is a late event occurring in 10% of MM patients and results in TP53 inactivation with poor prognosis.

Based on such karyotypic complexity, several attempts have been done to provide clues on the molecular basis of instability by using different approaches (gene expression analysis, DNA-based techniques, and deep genome sequencing). Zhan et al. [26] in 2006 first made a molecular classification, by identifying 7 subclasses of MM. In this model, the first class (MS class) was defined by the overexpression of the MMSET and/or FGFR3 genes resulting from translocation t(4;14). The second class (MF class) showed upregulation of MAF genes following translocations t(14;16) or t(14;20). The overexpression of CCND1 or CCND3, triggered by the translocations t(11;14) or t(6;14), identified the third and the fourth group CD1 and CD2, respectively. The fifth group (HY class) was represented by hyperdiploidy. The last two groups were characterized by a low incidence of bone disease with low levels of genes involved in bone disease (LB class), whereas the last group (PR class) was identified by high levels of genes involved in progression and proliferation. This molecular heterogeneity has been further confirmed and improved in several subsequent gene expression-based studies [27]. Moreover, copy numbers changes analysis by high-density single nucleotide polymorphism (SNP) array has identified other levels of molecular heterogeneity, which result in significant outcomes differences [28, 29]. Therefore, combining gene expression with copy number leads to more accurate analysis of this heterogeneity, which is related to the uncontrolled recombination mechanisms existing in this tumor. Remarkably, such knowledge can be exploited in both understanding MM biology and developing effective therapeutic strategies.

More recently, several efforts in deciphering molecular events driving MM progression have been made using genome sequencing analysis. This approach, by showing the complex subclonal structure of MM patients at diagnosis, which dynamically evolves over time, suggests the marked intertumor heterogeneity. Indeed, the mutational repertoire affecting genes of likely pathogenetic significance such as NRAS, KRAS, BRAF, p53, FAM46C, DIS3, SP140, LTB, ROBO1, and EGR1 indicates a cooperative role for multiple molecular pathways in supporting disease progression [17, 30–32]. Overall these studies demonstrate the existence of a multistep transformation process that changes MM genetic landscape over time (due to somatic mutations, epigenetic and chromosomal copy number variations).

Based on these findings, a clonal evolution has been proposed with progression disease achieved through branching, nonlinear pathways, which is a typical pattern of a complex ecosystem of clones competing for evolution [17, 33–35]. Therefore, all these studies suggest a disease landscape with complex pattern of genetic mutations at diagnosis aside from a Darwinian branching model of tumor evolution driving the alternating dominance of competing or collaborating clones present at diagnosis, over time. In such a scenario, the quantitative nature of next generation sequencing (NGS) data allows for higher resolution of the subclonal architecture of cancers and its monitoring over time with implication for prognostic stratification, tumor monitoring, and emergence of chemoresistance [17].

Overall, the tremendous knowledge achieved in MM molecular description with identification of high variability in its genomic architecture further underscores substantial heterogeneity of this hematologic malignancy and highlights the need for therapeutic interventions directed at multiple targets rather than a single genomic anomaly, as exemplified by success of combination therapies.

## 3. DNA Damage Response Mechanisms in MM Cells

Maintenance of genome integrity is crucial for tumor suppression and for the propagation of genomic information to subsequent generations. However, DNA integrity is persistently challenged by metabolism, errors in DNA replication and recombination, and exogenous genotoxic agents (ultraviolet light, oxidative stress, and chemical mutagens) that can lead to a range of DNA breaks. Indeed, these lesions can block genome integrity and if not repaired or repaired incorrectly lead to mutations or aberrations threatening cell viability. Thus, to counteract these attacks, cells use a sophisticate response system that, by inducing cell cycle arrest, allows DNA repair. Namely, to combat the constant threats posed to genome integrity, cells have evolved mechanisms—collectively termed the DNA damage response (DDR)—to detect DNA lesions and promote repair [36]. Such machinery is a complex and intertwined network of several proteins that enable proper DNA replication and that correct and repair breaches in the integrity and fidelity of the genetic code [37]. Cells defective in these mechanisms generally display an enhanced sensitivity towards DNA damaging agents and many of these defects cause human disease. Current studies have significantly increased our understanding on DNA damage response systems, allowing a better knowledge of such a complex feature orchestrated by tumor cells.

MM as well as most cancers has a striking genetic instability, which in turn leads to accumulation of mutational changes, some of which underlie tumor progression, drug resistance, and metastasis [19, 38]. Therefore the molecular basis causing this genetic diversity in cancer cells has important implications in understanding cancer progression. It is also noteworthy that most carcinogens operate by generating DNA damage and causing mutations [39]; consequently DNA repair provides a common mechanism for cancer-therapy resistance. A paradigm is the success of PARP inhibitors in those breast tumors, which lack functional BRCA1 or BRCA2 [40]. Namely, tumor cells with any DDR deficiency or “BRCAness” are likely to be particularly sensitive to PARP inhibitors because they are unable to cope effectively with the increase in lethal DSBs associated with replication fork collapse [41, 42]. However, BRCA-deficient tumors represent only a small percentage of cancer, restricting therefore the therapeutic utility of this synthetic lethal phenotype (SLI). In MM cells, direct evidence of homozygous loss or mutations in BRCA1/2 or other DDR genes is lacking, but an increased DNA repair activity capable of coping with a higher number of ongoing mutations has been previously reported. Overall, great progress has been made towards understanding the DDR but much remains to be learned.

**Figure 1:**
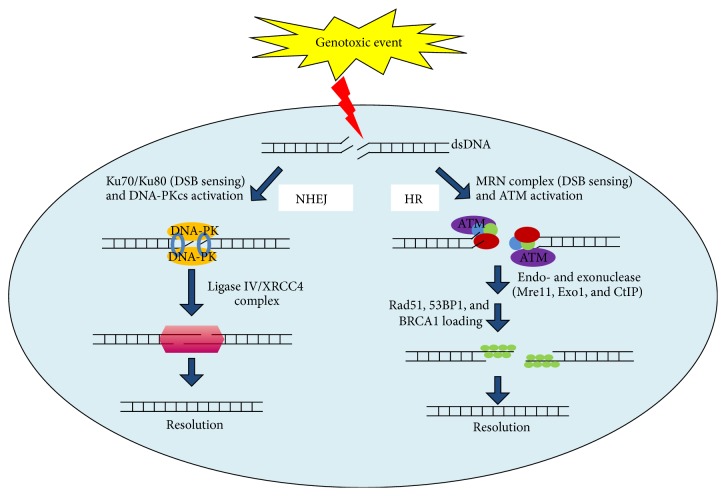
DSBs repair mechanisms. On the* left* part NHEJ. DSBs are identified by the ring-shape heterodimer Ku70/Ku80 which binds DNA broken ends and recruits the DNA-PKcs (DNA-dependent protein kinase catalytic subunit). This complex stabilizes DNA ends allowing a ligation carried out by XRCC4 and Ligase IV complex that finally reattaches the broken DNA. On the* right* part HR. The ATM kinase is recruited to DSB via an interaction with the MRN (Mre11-Rad50-Nbs1) complex, RNF8 and RNF168. Once ATM becomes activated, it phosphorylates multiple substrates including endo- and exonuclease (such as Mre11, Exo1, and CtIP) that are coated with ssDNA. Moreover ssDNA regions attract also Rad51 and other associated proteins (53BP1, BRCA1, etc.) which collectively assure new DNA synthesis. Defects of these mechanisms/cooperation lead to genomic instability, which in turn mediates tumor cell growth and progression.

In general, DDR mechanisms can be divided into single strand (SSB) or double strand (DDB) break repair, according to their specific activity. Namely, in presence of DNA single strand damage, the repair involves mismatch (MMR), base excision (BER), or nucleotide excision (NER) repair pathway. Importantly BER requires poly-ADP-ribose polymerase (PARP) which following DNA SSBs binding catalyzes synthesis and addition of large chains of poly-ADP-ribose (PAR) polymers on target proteins, including histones H1, H2B, and PARP1 itself [18]. If persistent or left unrepaired, SSBs result in potentially lethal double strand DNA breaks. Although DSBs do not occur as frequently as the SSBs lesions, they are difficult to repair and extremely toxic [43]. To handle this warning, cells employ several DSBs repair mechanisms: Non-Homologous end joining (NHEJ) and Homologous Recombination (HR) [44]. Whilst NHEJ is considered highly mutagenic pathway with its activity resulting in small insertions or deletions at the junction site, HR is error-free mechanism. NHEJ works primarily during G0-G1 phases of cell cycle. It promotes DNA broken ends bridging without using a specific template resulting therefore in a less accurate repair of DSB. Specifically, following DSBs Ku70/80 heterodimes binds DNA broken ends and recruits the DNA-PKcs (DNA-dependent protein kinase catalytic subunit). This complex stabilizes DNA ends allowing a ligation carried out by XRCC4 and Ligase IV complex [17]. By contrast, DSBs during S/G2 phase triggers repair activity via HR pathway, in which MRN complex acts as major player and the sister chromatid is used as template to copy the missing information into the broken locus. Such process begins with H2AX phosphorylation by PI3-K family members ATM (ataxia telangiectasia) and ATR (Rad3 related), after their recruitment to DSBs regions. Such event initiates a dynamic recruitment of MDC1 along with its binding partners (MRN complex, RNF8 and RNF168, etc.) at sites of DNA damage. Next a second wave involves proteins playing key roles in repair and maintenance of genomic integrity, including 53BP1 and BRCA1. Overall, a complex processes network preserves genome integrity in mammalian cells with its impairment that fuels instability (Figure 1).

In MM cells elevated HR activity supports the increased rate of mutation and progressive accumulation of genetic variation observed over time, as reported by Shammas et al. [45]. Likewise, also NHEJ impaired activity contributes to genomic instability of such a tumor. Specifically, defects in XRCC4 or Ku70 have been described in U266 and RPMI8226 cell lines by Herrero et al. [46]. These authors found an upregulation of both DSBs repair mechanisms in MM cells, suggesting that HR and NHEJ contribute equally to the enormous genomic instability featuring these cells. Genome sequencing analyses revealed mutations in several genes involved in these pathways including ATM, ATR, MRN complex, XRCC3-4, RNF168, and BRCA1 [17, 30, 31, 35, 47, 48] (Table 2). In line with these data, we have recently demonstrated that MM cells exhibit high levels of NAD^+^-dependent deacetylases SIRT6, which plays a key role in DSBs repair mechanisms and positively correlates with HR and NHEJ activities. We therefore propose this protein as crucial in preserving genome integrity of MM cells with its targeting as able to enhance chemotherapeutic response of DNA damaging Agents (Cea et al. manuscript submitted).

Overall, an imbalance between these two DSBs repair mechanism represents a hallmark of MM cells and contributes to its karyotypic instability.

## 4. Role of Epigenetic Changes and Telomeres in Genetic Instability of MM Cells

Likewise sequence alteration and chromosomal aberrations, also posttranslational processes, are common features of MM cells influencing gene expression and genome stability [18, 49, 50]. These are inheritable gene expression changes, named epigenetic process, which do not affect the genetic code [51]. Among such events, CpG islands methylation achieved by DNA methyltransferases (Dnmts), and histone modifications resulting from histone acetyltransferases (HATs) or histone deacetylases (HDACs), are frequently observed in MM cells [52, 53]. Indeed, genome-wide methylation microarrays have revealed specific changes in DNA methylation of MM cells according to clinical stages with progressive hypermethylation observed during disease progression [49, 54]. Of interest, since hypermethylation is associated with transcriptional silencing [55–57], whilst DNA hypomethylation is implicated in the genetic instability seen in many cancers [58], few authors suggest demethylating agents as alternative option to treat MM patients [30]. Importantly, 15–20% of MM patients harbor t(4;14) translocation, which results in increased expression of a histone methyltransferase gene, MMSET. Such event globally changes histone modifications, by supporting genomic instability in these patients [59–61]. Epigenetic changes in specific DNA damage repair genes have been also observed in MM patients as represented by the human thymine DNA glycosylate (TDG) that is involved in BER mechanisms. Methylation of this gene results in detrimental effect on DNA repair efficacy, further increasing genomic instability [62].

Also histone signature changes modulate gene expression and have been associated with cancer development. Specifically, HATs by catalyzing acetyl groups addition to lysine residues of histone tails allows relaxed chromatin state making DNA elements more accessible to transcription factors. On the other hand, HDACs, by removing acetyl groups from histone tails, make chromatin condensed and reduce activity of transcription factors [63]. Importantly, MM cells exhibit an imbalance of these two enzymes in favor of HDACs, which results in enhanced activity of key transcriptional factors and oncogenes [64–67].

A further regulator of genomic stability surveillance is represented by telomeres. These are nucleoprotein structures that cap the ends of chromosomes in order to prevent loss of genomic sequence during replication [39], and chromosomes fusion at the end [68]. Specific enzymes, named telomerase preserve telomere length and counterbalance their shortening following cell proliferation [69]. Opposite to normal cells, tumors reactivate telomerase, inducing tumorigenic phenotype [68]. Moreover, recent reports suggest one further mechanism of telomerase adopted by tumor cells to preserve telomere length, the alternative lengthening of telomeres (ALT) [70, 71]. Thus, based on these features, it is clear that although numerous mechanisms employed by tumor cells to maintain telomere length, it results critical for tumorigenesis and represents a cause or a consequence of genetic instability [18, 72, 73].

## 5. Clinical Implications of Genomic Instability in MM Cells

The constitutive ongoing DNA damage represents a trait of hematologic tumors [6], which leads to genomic instability and ultimately to more aggressive disease, often resistant to current therapies. Thus, genomic instability has important clinical implications with identified genomic alteration patterns providing basis for improved MM classification and prognostication. Example of such significance include the stimulated activity of DNA DSBs repair mechanisms NHEJ and HR in MM cells, in line with other hematologic malignancies. Indeed, hyperactivity associated with putative imbalance of these pathways observed in MM cells results in emergence of genetic changes responsible for disease progression and acquisition of drug resistances [46].

Whereas on one hand genomic instability is largely useful to transformed cells by providing a progressive growth advantage and development of drug resistance; on the other hand it may create exploitable vulnerabilities. Indeed, current therapeutic efforts aim to create synthetic lethal interactions in MM cells by targeting presumptive DNA repair defects of tumor cells specifically [74]. An example of this strategy is represented by marked sensitivity of MM cells to poly(ADP-ribose) polymerase (PARP) inhibitors triggered by 26S proteasome inhibition. Specifically this approach by impairing BRCA1 and RAD51 recruitment at DNA damaged sites makes a functional* BRCAness* state in MM cells. As such cotreatment with proteasome and PARP inhibitors does result in contextual synthetic lethality and leads to striking MM cell death [75]. A clinical phase I trial supporting efficacy of this strategy in MM patients is currently ongoing.

In addition to direct targeting genetic vulnerabilities, the acquired epigenetics knowledge has provided further valuable therapeutic insights as observed with recent using of HDAC and DNMTs inhibitors for the treatment of MM [76–82].

In summary, presumptive DNA repair defect(s) in complex pattern might result in selective sensitivity to certain classes of anti-MM therapeutics including DNA damaging agents, bortezomib and IMiDs.

## 6. Future Directions

The knowledge of biological MM features is evolving rapidly but much remains to be learned. It is a very largely heterogeneous disease but basis for its phenotypic and genomic multiplicity remains uncertain, though continued proteomic and sequence-level analysis of its architecture is likely to provide insight. Mechanisms whereby ongoing heterogeneity shapes tumor genomes still remains unclear. Future studies should clarify the nature of a possible defect in DSB repair (and, more broadly, in the DNA damage response), as well as the functionality of each repair-pathway component. Such expertise would be exploited to identify selective vulnerabilities created by underlying genomic instability, which may be tested by unbiased drug screens. Therefore, future efforts should focus not only on the identification of mechanisms causing genomic instability, but also on clinical translation of these information, which should eventually lead to new treatment options for patients with MM.

## Figures and Tables

**Table 1:** Recurrent chromosomal aberrations observed in MM and their prognostic relevance.

Aberration	Incidence	Outcome
Trisomies of chromosomes 3, 5, 7, 11, 15, 19, and 21	60%	Favorable
t(4;14)	15%	Poor
t(11;14)	20%	Favorable
t(14;16)	6-7%	Poor
del(17p)	8–10%	Poor

**Table 2:** Summary of molecules involved in DNA damage and frequencies of their mutations.

Genes	Walker et al. (*n* = 463)	Lohr et al. (*n* = 203)	Bolli et al. (*n* = 67)	Chapman et al. (*n* = 38)	^*∗*^Cifola et al. (*n* = 12)
ATM	18 (3%)	8 (3.9%)	2 (3%)	1 (2.6%)	4 (34%)
ATR	6 (1.3%)	2 (1%)	1 (1.5%)	0	3 (25%)
XRCC4	0	0	1 (1.5%)	0	0
RNF168	0	2 (1%)	2 (3%)	1 (2.6%)	0
BRCA1/2	0	2 (1%)	1 (1.5%)	1 (2.6%)	2 (34%)

^*∗*^Plasma cell leukemias.
